# Profiling of Host Cell Response to Successive Canine Parvovirus Infection Based on Kinetic Proteomic Change Identification

**DOI:** 10.1038/srep29560

**Published:** 2016-07-13

**Authors:** Hang Zhao, Yuening Cheng, Jianke Wang, Peng Lin, Li Yi, Yaru Sun, Jingqiang Ren, Mingwei Tong, Zhigang Cao, Jiawei Li, Jinliang Deng, Shipeng Cheng

**Affiliations:** 1 State Key Laboratory for Molecular Biology of Special Economic Animals, Institute of Special Animal and Plant Sciences, Chinese Academy of Agricultural Sciences, Changchun, 130112, China

## Abstract

Canine parvovirus (CPV) reproduces by co-opting the resources of host cells, inevitably causing cytotoxic effects to the host cells. Feline kidney F81 cells are sensitive to CPV infection and show disparate growing statuses at different time points post-infection. This study analysed the response of F81 cells to CPV infection at successive infection time points by iTRAQ-based quantitative proteomics. Differentially expressed proteins (DEPs) during 60 h of infection and at selected time points post-infection were identified by an analysis of variance test and a two-tailed unpaired *t* test, respectively. DEPs with similar quantitative changes were clustered by hierarchical clustering and analysed by gene ontology enrichment, revealing that 12 h and 60 h post-infection were the optimal times to analyse the autonomous parvovirus replication and apoptosis processes, respectively. Using the Metacore^TM^ database, 29 DEPs were enriched in a network involved in p53 regulation. Besides, a significantly enriched pathway suggests that the CPV-induced cytopathic effect was probably due to the deficiency of functional CFTR caused by CPV infection. This study uncovered the systemic changes in key cellular factors involved in CPV infection and help to understand the molecular mechanisms of the anti-cancer activity of CPV and the cytopathic effects induced by CPV infection.

Canine parvovirus (CPV) is a member of autonomous parvoviruses in the *Parvoviridae* family. It appears as an icosahedral capsid that encloses a single-strand DNA genome that is 5.2 kb long. CPV replicates without the use of a helper virus and hence is called autonomous, but it utilises the machinery and components of host cells, including DNA polymerase and RNA polymerase ΙΙ for DNA replication and RNA transcription, respectively. Consequently, CPV replication is restricted to the S phase of the cell cycle^1^,^2^.

The CPV genome contains two open reading frames; by alternative splicing, one produces two mRNAs encoding two non-structural proteins (NS1 and NS2), and the other transcribes two mRNAs encoding two structural proteins (VP1 and VP2)^3^. The VP1 and VP2 proteins contain the most important antigenic epitopes, which are targeted by neutralising antibodies. VP2, which represents 90% of the viral capsid, also functions as a viral ligand that determines the viral host specificity and tissue affinity^4^. NS1, a pleiotropic phosphoprotein, is thought to be a culprit of apoptosis of CPV-infected cells. For instance, NS1 of CPV-2 has been shown to induce caspase-dependent and p53-independent apoptosis^5^.

CPV quickly spread worldwide within months of identification and now threatens various species hosts^6^. CPV infection causes high fatality in neonatal animals and severe haemorrhagic enteritis in adult dogs^7^. It is introduced by faecal-oral transmission, and it initially infects and replicates in the rapidly dividing cells of lymphoid tissues, intestinal crypt epithelial cells, and precursor cells in the bone marrow. Severe haemorrhagic enteritis increases the risk for viral translocation and coliform septicaemia, leading to septic shock and ultimately death^8^. CPV infects cells by binding to the transferrin receptor^9^, and CPV pathogenicity is thought to be caused by the non-structural proteins of parvoviruses^10^.

Although infection and replication of parvoviruses kill host cells, the extent of cell death strongly depends on the type of the host cell. For instance, neoplastic cells are preferentially killed over normal cells^11^,^12^. Due to this characteristic, a rodent parvovirus has been used in a phase Ι/ΙΙa clinical trial to prevent tumour recurrence in patients with glioblastoma multiforma^5^. Currently, the clinical application of several chemotherapeutic agents is limited because of their severe toxic effects to healthy cells. Anti-cancer therapy using a virus that can selectively target cancer cells has become a popular approach. CPV is a virus that can be potentially used to treat cancer^13^. A previous study has shown that CPV2 NS1 can lead to regression of skin tumours in Wistar rats without producing toxic side effects on healthy cells^14^. Moreover, the CPV NS1 protein shows anti-tumour activity in a mouse mammary tumour model, and it further stimulates the immune cells to attack the tumour^15^.

Proteomic based approaches have been widely used to develop extensive cellular protein databases that focus on infection by viruses^16^,^17^. However, no such study has been conducted to understand the molecular mechanisms involved in host response to CPV infection. Isobaric tag for relative and absolute quantitation (iTRAQ) is an *in vitro* labelling method that has the capability to compare several time points during a single experiment^18^. The method is considered more sensitive than difference gel electrophoresis, and it has been demonstrated to be effective and accurate in characterising numerous diseases^19^,^20^. In this study, we utilised an iTRAQ-labelled mass spectrometry approach to explore the proteomic changes of F81 cells under different cellular growth behaviour induced by CPV infection. Our findings will help to reveal the biological processes of different infection stages and systematically understand the host response under CPV infection.

## Results

### General experimental design and overview of the quantitative proteomic profile of F81 cells

**Figure 1:**
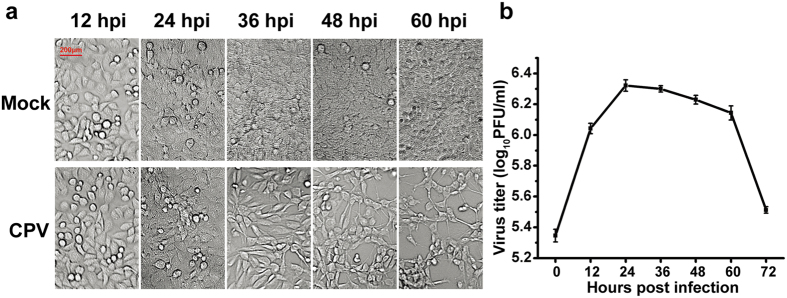
CPV infection of F81 cells. (**a**) Cytopathic effects of CPV infection. F81 cells were infected with CPV at a MOI of 1. The different panels show the cytopathic effects at the indicated time points after infection. Images were taken at an original magnification of 20×. (**b**) One-step growth curve of the CPV-HLJ-6 strain in F81 cells.

The F81 cells were selected because they efficiently support CPV replication and show specific cytopathic effects (CPEs)^21^,^22^,^23^,^24^. The cells were infected at a multiplicity of infection (MOI) of 1 and analysed between 0 and 60 h post-infection (hpi). As shown in Fig. 1a, the mock-infected cells maintained growth without obvious CPEs. In contrast, CPEs in the infection groups became noticeable at 36 hpi and progressed thereafter, and more than half of the cells had become detached at 60 hpi. As shown in Fig. 1b, the viral titre reached a plateau between 24 hpi and 60 hpi, and then rapidly declined. Using the dynamic changes in CPEs and viral titre as a guide, time points between 12 hpi and 60 hpi were selected for comprehensive proteomic analysis during which the viral titre remained high while the CPEs progressed in the infected cells.

**Figure 2:**
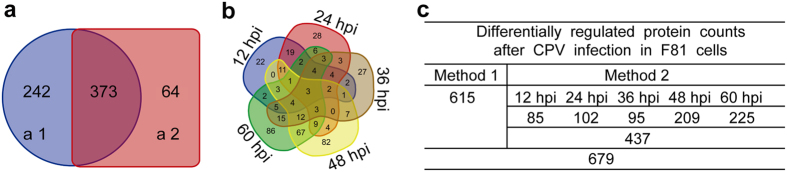
Summary of differentially expressed proteins (DEPs) identified after CPV infection in F81 cells. (**a**) Venn diagram of DEPs between groups (a1) and (a2). (a1) Proteins identified over the entire infection process. (a2) Summary of DEPs identified at each time point. (**b**) Overlap between DEPs identified at each time point. (**c**) Counts of differentially regulated proteins.

Based on a combination of three biological replicates from two mocks and five infected samples, the iTRAQ analysis identified a total of 3,224 proteins. Proteins were designated as differentially expressed proteins (DEPs) if they had expression fold-change ratios ≥1.2 or ≤0.833 and the *p*-value was less than 0.05. By two statistical methods, we identified 615 DEPs during the entire infection process by method 1 and 437 DEPs for all five time points by method 2 (Fig. 2c). As shown in Fig. 2a1,a2, a total of 679 DEPs were identified by combining the two selection methods, which was greater than the number identified by each of the individual methods. Among the 679 DEPs, 373 proteins were identified by both methods.

Among the 437 DEPs identified by method 2, the expression of 85, 102, 95, 209, and 225 proteins was significantly changed at 12, 24, 36, 48, and 60 hpi, respectively (Fig. 2b). As shown in Fig. 2b, only 3 of 437 proteins were identified at all five time points. Moreover, 22, 28, 27, 82, and 86 DEPs were separately identified at the each of the five time points, respectively. These proteins were the key proteins used to study the biological processes at certain time points.

A summary of the 615 quantitatively DEPs obtained by method 1 are shown in Supplementary Table S1. The quantitatively DEPs selected at each time point by method 2 are listed in Supplementary Tables S2.1–2.5, respectively. The abbreviations of all 679 differentially expressed proteins are listed in Supplementary Table S3; these abbreviations appear in the figures and their corresponding descriptions.

### Biological processes of DEPs at 12, 24, 36, 48, and 60 hpi were enriched by DAVID software

To analyse the potential functions of the significantly DEPs at each time point, we then performed a gene ontology enrichment of biological processes using DAVID software for the proteins obtained by method 2. Generally, gene ontology terms are enriched by David software without fuzzy heuristic clustering. So, an overwhelming number of terms were generated, which were too difficult to analyse. In order to reduce the redundant terms, we re-analysed them by “Functional Annotation Clustering,” and only the term with the minimum *p*-value in each cluster was reserved. As a result, 60/79, 57/77, 22/33, 119/166, and 91/135 of the biological process terms at each time point were removed.

**Figure 3:**
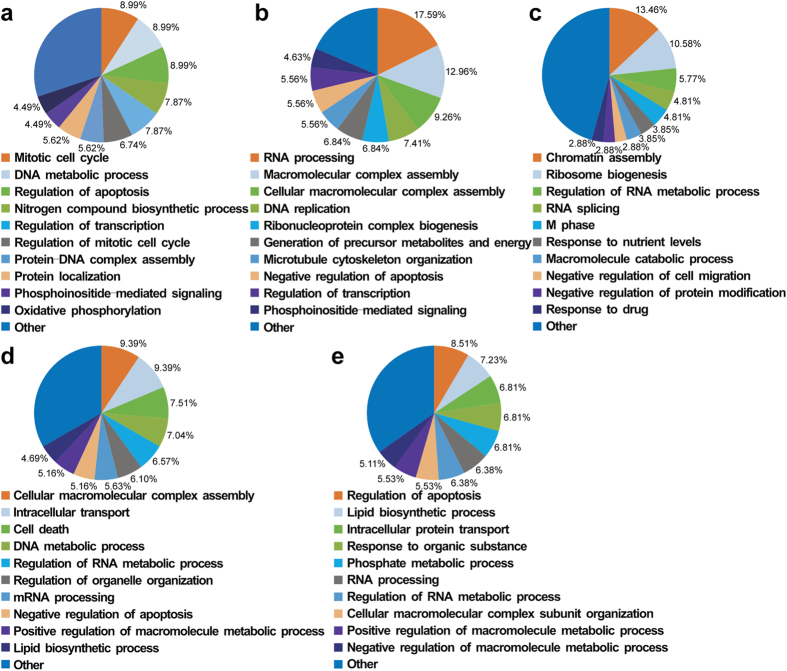
Gene ontology (Biological Process) analysis of differentially expressed proteins at (**a**) 12 hpi, (**b**) 24 hpi, (**c**) 36 hpi, (**d**) 48 hpi, and (**e**) 60 hpi. Note that at early infection, the majority of the altered proteins are involved in the cell cycle; while at late infection, the majority of the altered proteins are involved in regulating apoptosis.

As shown in Fig. 3a–e, the retained functions of these proteins at each time point are depicted. Enriched functions of the DEPs at 12 hpi were mainly related to the cell cycle, including regulation of the mitotic phase of the cell cycle. At 24 hpi, numerous proteins involved in RNA processing were enriched. By 36 hpi, the DEPs were significantly enriched in proteins related to chromatin assembly (*p* = 1.88E-14). At 48 hpi, enriched functions were related to cellular macromolecular complex assembly (*p* = 3.91E-08) and regulation of organelle organisation (*p* = 2.99E-05). The functional terms in the regulation of apoptosis were enriched at each time point; but at 60 hpi, proteins involved in the regulation of apoptosis became the largest component (8.51%) of all DEPs.

### Proteins involved in the stimulus-response and regulation of apoptosis were enriched by Gene Cluster 3.0 and DAVID software

**Figure 4:**
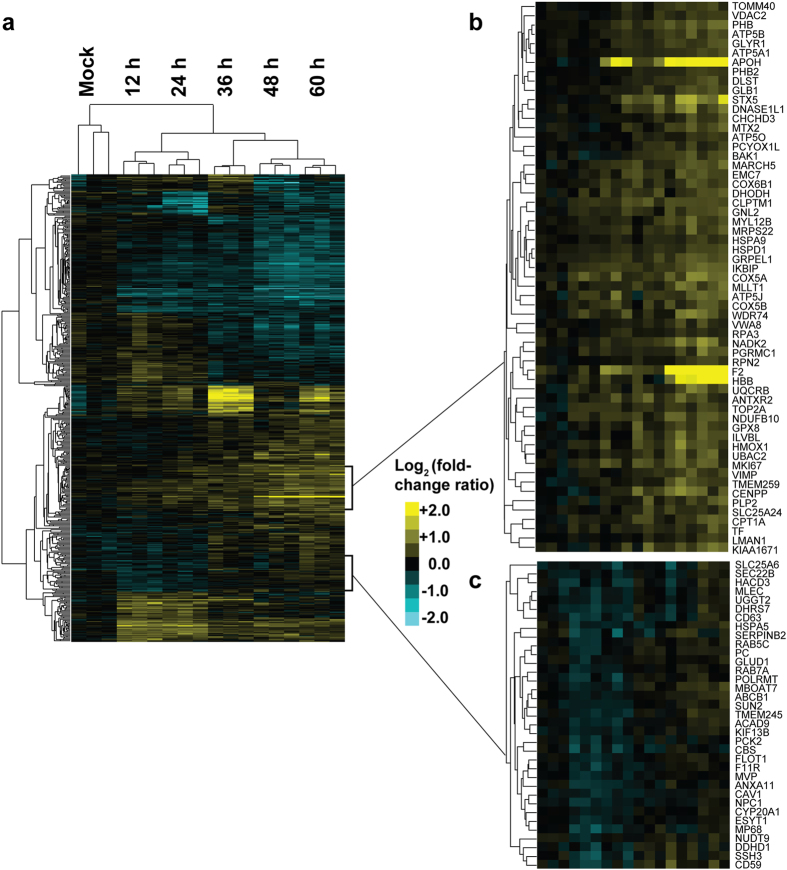
Hierarchical cluster analysis of 615 differentially regulated proteins. (**a**) The results of mock-infected and CPV-infected cells at the indicated time points after infection. (**b**) Specific enlargement of cluster A. (**c**) Specific enlargement of cluster B. The proteins in cluster A are mainly involved in regulating apoptosis, and the proteins in cluster B are mainly involved in the stimulus-response.

As shown in Fig. 4a, hierarchical clustering of the *Felis catus* proteins was generated using the DEPs listed in Supplementary Table S1. The analysis revealed that all replicates clustered tightly. Interestingly, the DAVID software analysis showed that most proteins in a given cluster were enriched for functions involved in similar biological processes.

As shown in Fig. 4a, most clusters were enriched in the biological processes of energy metabolism, the cell cycle, translation, and RNA-related functions. Both cluster A (Fig. 4b) and cluster B (Fig. 4c) are highlighted. Sixty-nine proteins in cluster A were up-regulated during the whole infection period, especially at 48 hpi and 60 hpi. The DAVID analysis indicated that the proteins in cluster A were mainly associated with cellular apoptosis, including HMOX1, TOP2A, HSPA9, HSPD1, F2, HBB, SLC25A24, BAK1, VDAC2, PHB, APOH, and VIMP. Moreover, five of these proteins were classified as negative regulators of apoptosis.

Furthermore, in cluster B consisting of 35 proteins, 10 are known to be stimulus-responsive, 7 of which are additionally known to respond to viral infections. For instance, both RAB7A and CD63 are required for the HIV-1 replication cycle^25^,^26^. In addition, LSD1, HSPA5, and CAV-1 have been reported to influence the efficiency of viral infection and replication^27^,^28^,^29^,^30^,^31^; NPC1 is the key influencer for Ebola virus to enter cells^32^; SEC22b can inhibit the release of infectious viral progeny^33^; and the down-regulation of CD59 is associated with the development of hepatitis B cytotoxicity^34^.

### A network focusing on p53 regulation and a pathway involving cystic fibrosis transmembrane conductance regulator (CFTR) degradation were enriched according to the Metacore^TM^ database

**Figure 5:**
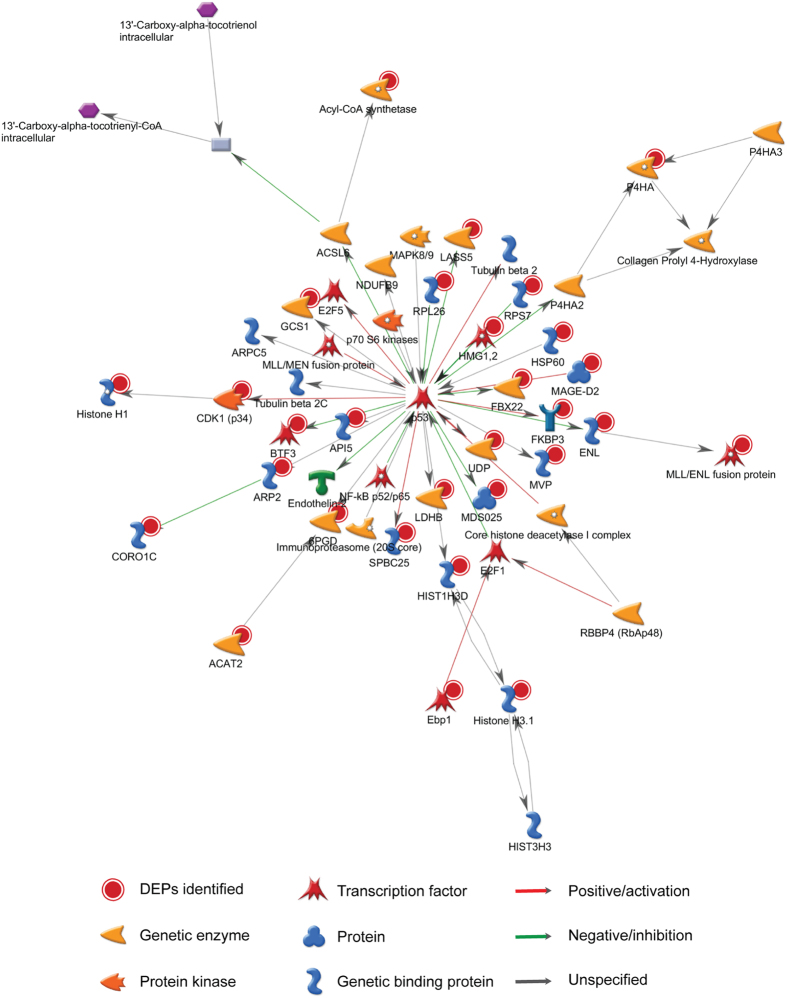
Twenty-nine differentially expressed proteins (DEPs) enriched in the network that are closely related to the regulation of p53. This network was enriched by 615 DEPs, with a *p*-value of 1.130E-49. The proteins highlighted by red points were the DEPs identified in this study.

Using Metacore^TM^ software, 29 DEPs associated with p53 were significantly enriched (*p*-value = 1.130E-49), including BAK1, VDAC2, HSP60, and Ebp1 (Fig. 5), indicating that CPV infection may trigger biological processes of p53 regulation.

**Figure 6:**
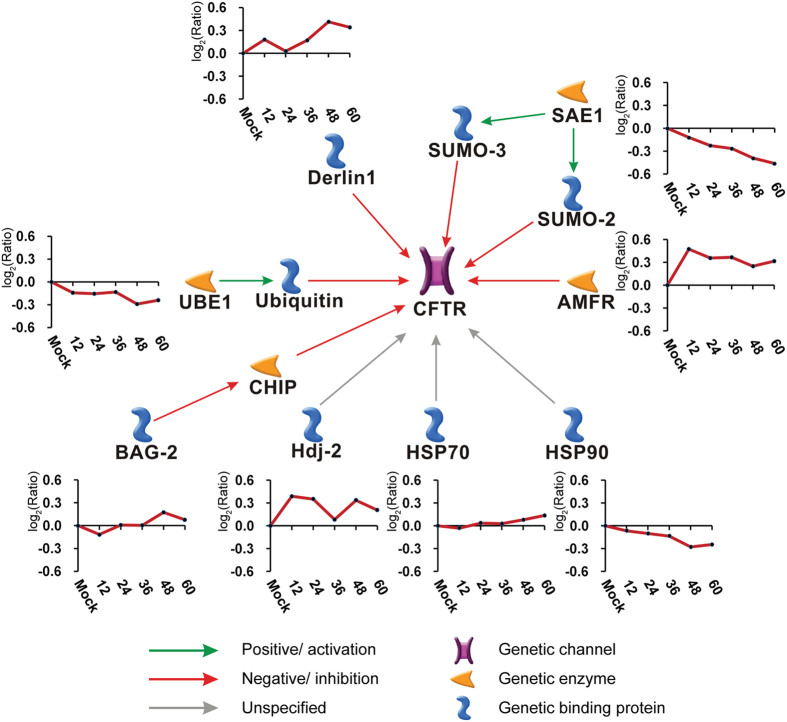
Eight differentially expressed proteins (DEPs) enriched in the pathway that are involved in the degradation of CFTR. This pathway was enriched among the 615 DEPs, with a *p*-value of 9.398 E-06. The expression trends of eight DEPs are shown in the corresponding graphs. The y-axis shows the relative abundance of each protein, and the abundance was calculated by the log_2_ (fold-change ratio) of each time point.

The pathways were enriched and ranked according to their *p*-values by the Metacore^TM^ database (Supplementary Table S4). These pathways are involved in processes of cell cycle regulation, cytoskeleton remodelling, cell apoptosis and survival, oxidative phosphorylation, and immune response. Notably, eight DEPs were enriched in the pathway involved in the “CFTR folding and maturation” with a *p*-value of 9.398E-06 (Supplementary Fig. S1). The detailed analysis depicted in Fig. 6 indicates that eight DEPs were directly or indirectly associated with the CFTR (Derlin 1, AMFR, HSP70, HSP90, Hdi-2, UBE1, BAG-2, and SAE1). Among them, Derlin 1 and AMFR were up-regulated during infection, which can directly degrade the abnormal CFTR^35^,^36^.

### iTRAQ-MS data were verified by western blotting

**Figure 7:**
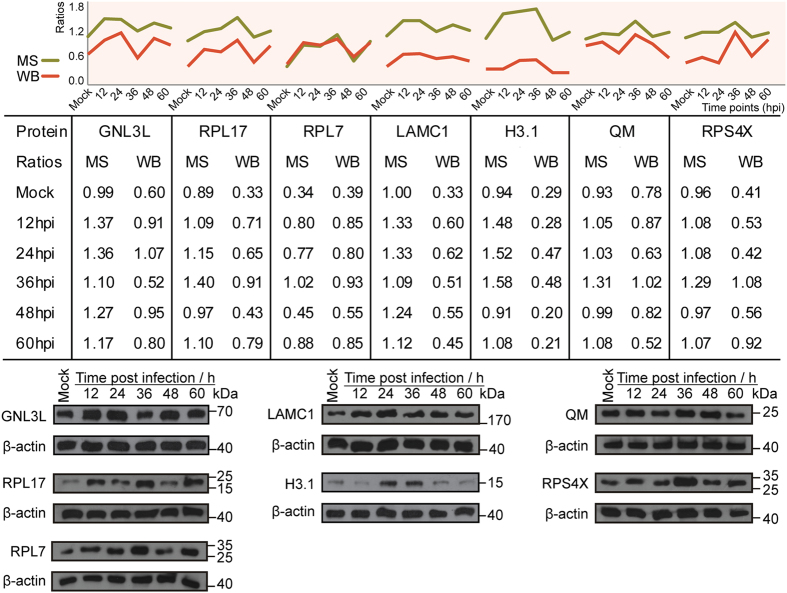
Comparison of seven protein changes identified by mass spectrometry and western blot. Verification of the expression of GNL3L, RPL17, RPL7, LAMC1, H3.1, QM, and RPS4X in mock-infected cells and CPV-infected cells at 12, 24, 36, 48, and 60 hpi by western blot analysis; β-actin was used as a loading control. The mass spectrometry fold-change ratios of each protein at each time point were consistent with the ratio values of the western blot band intensities analysed by Gel-Pro Analyzer 4 software.

The verification of DEPs by western blotting was limited by the availability of antibodies to *Felis catus* proteins. Initial tests selected a set of proteins that were significantly changed during infection and also reliably cross-reacted with antibodies to the corresponding human protein. As shown in Fig. 7, the western blots confirmed the differential expression of ribosomal protein S4X (RPS4X), 60S ribosomal protein L7 (RPL7), guanine nucleotide-binding protein-like 3-like protein (GNL3L), laminin subunit gamma-1 (LAMC1), histone H3.1 (H3.1), 60S ribosomal protein L17 (RPL17), and 60S ribosomal protein L10 (RPL10 or QM) detected by iTRAQ.

## Discussion

In this study, we used proteomics to investigate the global profiles of differentially expressed cellular proteins under CPV infection. The features of our experimental design and data analysis are as follows: 1. The kinetic changes in the expression levels of cellular proteins along the infection course, consisting of five different time points, were quantified, which represented an improvement over a cross-sectional picture if only a sample from a single time point was analysed; these results provided us a kinetic view of the changed expression of cellular proteins under the cytopathic insult of CPV infection. 2. Two mocks were analysed. These two mocks helped to distinguish the natural kinetics of the cellular protein expression and helped to set reasonable standards to screen the DEPs. 3. Three biological replicates were prepared for each sample to assess inter-experimental variations. 4. The DEPs identified over the whole infection process were determined to be significant by an analysis of variance test, while those at each of the five time points were determined to be significant by the two-tailed unpaired *t* test.

As expected, a large amount of proteomic data was generated in this study. Venn diagramming functional annotation, hierarchical clustering, and pathway enrichment were utilised to analyse the results. A common analysis strategy in proteomics is to look for a period in which the changes in the protein expression were the most prominent. However, such a strategy is dissociated from the status of viral infection and the impact on the infected cells. A different strategy in this study was employed that combined the detected significant changes in DEPs with viral replication and CPEs. In view of the CPEs and the one-step growth curve shown in Fig. 1b, the viral titre was increased quickly during the first 12 hpi. In addition, the DEPs involved in the mitotic cell cycle were significantly enriched by up to 6.74% of the total DEPs at 12 hpi (Fig. 3). Since the cells had efficient proliferation at 12 hpi, this time point was suitable for studying the cellular proteins involved in autonomous parvovirus replication.

As shown in Fig. 4c, the proteins involved in the stimulus-response were enriched, and most of them are reported to respond to viral infection. These proteins can be potential targets for controlling viral infection. Dynamic changes in the expressed proteins in cluster B were noted. They were initially down-regulated at 12 and 24 hpi, followed by a rapid return to near basal levels at 36 hpi and slight up-regulation at 48 and 60 hpi. Thus, 12 and 24 hpi were the best time points to study these proteins (Fig. 4).

Similarly, 60 hpi was the optimal time point to analyse the DEPs involved in the regulation of apoptosis because apoptosis-related proteins accounted for the largest percentage at this time point (Fig. 3e), and the CPEs were the most serious at 60 hpi (Fig. 1b). As shown in Fig. 4b, many proteins related to cell apoptosis were enriched in cluster A, and they were up-regulated during the entire infection period, but most strikingly at 48 and 60 hpi. Thus, we concluded that the late stage of CPV infection was a better time to investigate the CPV-induced F81 cell apoptosis process. As apoptosis may be the main mechanism utilised by viral oncolytic therapy, these identified DEPs may be useful for detailed studies in the future.

The anti-cancer activity of CPV is closely related to its apoptosis-inducing effect. A previous study has shown that CPV2 NS1 induces apoptosis mainly through the mitochondrial pathway^37^. Coincidently, we found that BAK1 was up-regulated at 48 hpi and 60 hpi in this study. BAK is a pro-apoptotic regulator that plays a central role in mitochondrial membrane permeabilisation^38^. It suppresses tumourigenesis by mediating apoptosis of tumour cells^39^. For example, BAK over-expression can effectively induce apoptosis in gastric cancer cells^40^. Also, elevations in BAK protein levels have been shown to accelerate apoptosis in murine lymphoid, lung cancer, and breast cancer cells^41^. In addition, VDAC2, which can recruit newly synthesised BAK to the mitochondria to carry out apoptosis^42^, was also up-regulated at 48 hpi and 60 hpi in our study. These findings remind us that the anti-cancer activity of CPV may be closely related to the over-expression of BAK1 and the mitochondrial apoptotic pathway that is mediated by it. Lakshman Santra *et al*. suggest that CPV2 NS1 might be effective against tumours with the p53 mutation^43^. As a critical effector of apoptosis, BAK can participate in the apoptotic process without activation of p53. Therefore, over-expression of the *bak* gene may be a new strategy for the treatment of tumours, especially for tumours bearing p53 mutations.

What’s more, we enriched a panel of 29 proteins that are associated with p53 by Metacore^TM^ software, as shown in Fig. 5. As p53 is known as an important anti-cancer protein, these proteins could be related to cancer development. For instance, API5 was enriched in this network, and the API5 expression level was reduced in F81 cells at 48 hpi and 60 hpi. API5 is an apoptosis inhibitory protein that can suppress E2F-dependent apoptosis^44^. It is frequently up-regulated in tumour cells and functions as a master regulator of immune escape genes in tumours^45^. Based on these findings, CPV may be a powerful apoptosis-modulating agent that could down-regulate API5 gene expression.

We found that the HSP60 expression level was increased at 60 hpi. Heat shock proteins (HSPs) are a group of proteins that respond to stress, including heat, hypoxia, irradiation, infection, or toxic agents^46^. HSP has been shown to influence apoptosis in tumour cells, regulate p53 function, and induce an immune response to tumours^47^. Increased HSP levels make cells more resistant to apoptosis, but they also can increase immunity to cancer^48^. For example, over-expressed HSP60 protein is suggested to promote prostate cancer, colorectal cancer, and primary breast cancer; for these cancers, HSP60 inhibitors may be used as anti-cancer agents^49^,^50^,^51^. Whereas, a loss of HSP60 immunopositivity is related to the development and progression of bronchial cancer in smokers with chronic obstructive pulmonary disease^52^. In this experiment, we also identified that the HSP70 expression level was increased at 60 hpi and the HSP90A expression level was decreased at 48 hpi and 60 hpi. As the influence of the HSP expression changes due to different cancers, the anti-cancer significance of the differential expression of HSPs in CPV-infected cells is complicated and requires further study.

Previous studies have shown that the CPV NS1 protein exhibits anti-tumour activity in mammary tumours and skin tumours^13^,^15^, but the complete mechanisms of its anti-cancer activity have not been revealed. In this study, we first identified a group of cancer-related proteins by a proteomics technique. Some of these proteins were not noted for CPV-induced apoptosis or anti-cancer function, which would provide a new perspective for studying the anti-cancer mechanisms of CPV.

Using Metacore^TM^ software, we were able to suggest the main pathways related to CPV infection. Interestingly, a pathway that involved the degradation of CFTR was significantly enriched in our study. CFTR is a transmembrane protein that functions as an ATP-gated chloride channel^53^. Deficiency in CFTR can lead to several diseases related to the reduction of chloride secretion and the increase of sodium uptake^54^. Many studies have suggested that wound healing function is impaired in CFTR-deficient epithelial cells and that CFTR may be effective in cell repair after injury^18^,^55^,^56^. CPV is normally cytopathic in infected F81 cells. Typical CPEs appear as a mesh of monolayer cells prompted by detached cells. These results provided us hints that CPV may trigger CFTR deficiency and influence the cell pathological change by reducing the wound healing of infected cells.

In summary, DEPs and their expression kinetics in CPV-infected F81 cells were characterised with iTRAQ analysis. There were dynamic changes in DEPs over the course of infection. DEPs that are involved in viral defence or apoptosis and are related to cancer may be useful for studying anti-viral and oncolytic therapy. Finally, the identified DEPs that are involved in folding and maturation of CFTR provide us with a list of candidates for further understanding the pathogenesis of CPV-infected host cells.

## Experimental Procedures

### General experimental design

CPV-HLJ-6 (GenBank accession no. KR611475.1) was passaged ten times in F81 cells, resulting in a final stock containing viruses at 10^5.5^ TCID_50_/mL. For infection, 10^6^ F81 cells were plated in 25 cm^2^ culture flasks and, at the same time, inoculated by CPV at a MOI of 1. Infected cells were collected at 12, 24, 36, 48, and 60 hpi, and uninfected mock-treated cells were collected at 0 and 48 h after plating. All experiments were repeated in three biological replicates with cells at different passage numbers.

### Protein extraction and quantification

The collected cells were lysed by Protein Extraction Reagent (Thermo Scientific™), containing a protease inhibitor cocktail, for 10 min under gentle shaking conditions. The samples were centrifuged at 15,000 *g* for 10 min, and the supernatants were collected. The protein concentrations were measured by the Bradford protein assay.

### iTRAQ labelling and peptide fractionation

The iTRAQ labelling was conducted according to the protocol of the iTRAQ reagents (8 plex, Applied Biosystems). Briefly, the protein samples were thermally denatured, reduced, and alkylated prior to trypsin (Promega) digestion for 15 h. The digested samples were labelled with each component of the iTRAQ reagent kit: two mock samples were labelled with 113 and 114; and five CPV-infected samples collected at 12, 24, 36, 48, and 60 hpi were labelled with 115, 116, 117, 118, and 119, respectively. Three biological replicates were prepared for all samples. Next, an equal ratio of each set was pooled for analysis.

Additionally, to reduce the complexity of the peptide mixtures, reverse-phase chromatography using a RIGOL L-3000 system was applied for separating the peptides. The labeled peptide mixtures were dissolved in 100 μL of mobile phase A [2% acetonitrile in ddH_2_O, pH 10] and centrifuged at 14,000 *g* for 20 min prior to loading onto the column. Samples were eluted by injection of stepwise gradients of mobile phase B [2% ddH_2_O in acetonitrile, pH 10], with a flow rate set at 700 μL/min. Each fraction was eluted for 1.5 min.

### Analysis by Q-Exactive mass spectrometry

The fractionated peptide mixtures were analysed by a Q-Exactive mass spectrometer equipped with an EASY-nLC 1000 System (Thermo Fisher Scientific). The spray voltage, capillary temperature, and declustering potential of the source ionisation parameters were set as 2.1 kV, 250 °C, and 100 V, respectively.

A Top 20 data-dependent acquisition mode was selected in the mass spectrometer, with automatic switching between MS and MS/MS. Full-scan MS mode (350−1800 *m/z*) was operated at a resolution of 70,000, with an automatic gain control target of 1 × 10^6^ ions and a maximum ion transfer of 60 ms. The precursor ions were fragmented by high-energy collisional dissociation and subjected to MS/MS scans with the following parameters: resolution, 17,500; automatic gain control, 5 × 10^6^ ions; maximum ion transfer, 70 ms; intensity threshold, 5,000; and normalised collision energy, 29%.

### Database searching and protein quantitation

All raw data files generated by mass spectrometry were analysed by Proteome Discoverer software 1.3 (Thermo Fisher Scientific) using the Mascot search engine against the *Felis catus* protein sequences downloaded from the NCBI reference sequences (Refseq) database (downloaded on March 20, 2015, containing 35,247 entries). The parameters for database searching were as follows: trypsin was selected as the enzyme; up to two missed cleavages were allowed; the precursor and fragment ion mass tolerance were set to 15 ppm and 20 mmu, respectively; carbamidomethylation on cysteine was set as a fixed modification; oxidation on methionine and iTRAQ 8 plex labels at the N-termini and at lysine side chains were allowed as dynamic modifications. The strict maximum parsimony principle was applied, and only peptide spectrum matches with high or medium confidence were considered for protein grouping. Ion peaks were integrated based on the most confident centroid with a tolerance of 20 ppm.

The quantification was also performed by Proteome Discoverer software 1.3, which can automatically calculate the relative abundance of iTRAQ-labeled peptides as well as the corresponding proteins. Proteins identified with two or more unique peptides were considered as a highly confident identification and used for quantification. Also, to ensure the accuracy of quantification, only the proteins whose coefficient of variation of three biological repeats were less than 20% were identified as DEPs. The protein ratios were calculated from channels (114/113 to 119/113) for all proteins identified in each iTRAQ experiment. Two identical mock samples, with iTRAQ labels of 113 and 114, were used as references. The fold threshold for changed protein fold-change ratios was set at ±1.20, which covers a 95% quantification area based on the normal distribution of two mock samples (113 versus 114) in all biological replicates^57^. Thus, the proteins with fold-change ratios ≥1.20 or ≤0.83 were selected as DEPs.

To identify the DEPs more comprehensively, we used two methods: An analysis of variance test that included a *p*-value < 0.05 and at least one of the fold-change ratios between any time points passing the threshold was used to identify the DEPs of all groups (Method 1). Furthermore, to clarify the protein alterations of each time points, a two-tailed unpaired *t* test that included a *p*-value < 0.05 and a fold-change ratio between a given time point and the mock (114) passing the threshold was used for screening the DEPs at the different time points (Method 2).

### Bioinformatics data analysis

The majority of the proteins in the *Felis catus* database are not annotated with biological functions. Nevertheless, because most of the identified proteins shared high homologies to *Homo sapien* proteins, we blasted DEPs defined by iTRAQ analysis with the *H. sapien* protein database in Swissprot. Only the proteins with at least 70% identity were considered to be matched.

The DEPs were submitted to the online software DAVID (http://david.ncifcrf.gov) for enrichment analysis of the gene ontology category “Biological Process”. Redundant items were reduced by “Functional Annotation Clustering” with medium classification stringency. The terms with the minimum *p*-values in every cluster were then selected out and reserved.

The patterns of differential protein expression were displayed by hierarchical clustering according to the fold-change ratios. The protein relationships were calculated by average linkage with uncentered correlation using Gene Cluster 3.0.

The network analysis of the DEPs was performed by the “Analyze Network Algorithm” provided by Metacore^TM^ software. The pathway analysis of the DEPs was performed using the GeneGo pathway maps in the Metacore^TM^ database (version 6.24; build 67895, Thomson Reuters). In addition, the Pathway Maps tool was used for pathway enrichment evaluation, in which the *p*-values were calculated based on the hypergeometric distribution with the default background database.

### Western blot analysis

Cells were harvested and cellular proteins were extracted as mentioned above. Total cellular proteins from different time points were separated by SDS-PAGE and electrotransferred to nitrocellulose membranes (Whatman). The membranes were blocked with 5% non-fat milk powder dissolved in tris-buffered saline, containing 0.05% Tween-20, for 2 h at room temperature. Primary antibodies (see below) were diluted 1:500 in blocking buffer and incubated with the membranes overnight at 4 °C. Next, the membranes were incubated with the appropriate secondary antibodies diluted in blocking buffer at 1:3000 for 2 h at room temperature. Detected proteins were revealed using the ECL Detection Kit (KeyGen Biotech).

The following rabbit antibodies were used: Ribosomal Protein S4X Polyclonal Antibody (YT4135), Ribosomal Protein L17 Polyclonal Antibody (YT4098), QM Polyclonal Antibody (YT3916), Histone H3.1 Polyclonal Antibody (YT2163), Laminin γ-1 Polyclonal Antibody (YT2531), GNL3L Polyclonal Antibody (YT1938), Ribosomal Protein L7 Polyclonal Antibody (YT4118), and β-Actin Polyclonal Antibody YT0099). All the rabbit antibodies were purchased from ImmunoWay Biotechnology Company. The Peroxidase AffiniPure goat Anti-Rabbit IgG was used as the secondary antibody (Jackson ImmunoResearch Lab.).

## Additional Information

**How to cite this article**: Zhao, H. *et al*. Profiling of Host Cell Response to Successive Canine Parvovirus Infection Based on Kinetic Proteomic Change Identification. *Sci. Rep.* **6**, 29560; doi: 10.1038/srep29560 (2016).

## Supplementary Material

Supplementary Information

Supplementary Tables
