# Supplementary material for: Profiling of Host Cell Response to Successive Canine Parvovirus Infection Based on Kinetic Proteomic Change Identification
Source: Sci Rep. 2016 Jul 13;6:29560. doi: 10.1038/srep29560 (PMC4942776; doi:10.1038/srep29560)

**Supplementary Information**

# Profiling of Host Cell Response to Successive Canine Parvovirus Infection Based on Kinetic Proteomic Change Identification

Hang Zhao, Yuening Cheng, Jianke Wang, Peng Lin, Li Yi, Yaru Sun, Jingqiang Ren, Mingwei Tong, Zhigang Cao, Jiawei Li, Jinliang Deng & Shipeng Cheng*

1. **Supplementary Table S1.** List of differentially expressed proteins during the whole course of CPV infection.
2. **Supplementary Tables S2.1 to S2.5.** List of differentially expressed proteins in F81 cells at 12, 24, 36, 48, 60 h post CPV infection, respectively.
3. **Supplementary Table S3.** List of abbreviations for 679 differentially expressed proteins.
4. **Supplementary Table S4.** The pathways enriched by the Metacore™ database.
5. **Supplementary Figure S1.** The pathway involved in "CFTR folding and maturation," which was enriched by Metacore™ software.

**Figure S1.** The pathway involved in “CFTR folding and maturation” which was enriched by Metacore™ software. The red and blue bars, numbered 1–5, represent the relative abundances of the corresponding proteins at 12, 24, 36, 48, and 60 h post CPV infection, respectively. The abundances are calculated by $\log_2$(fold-change ratios).

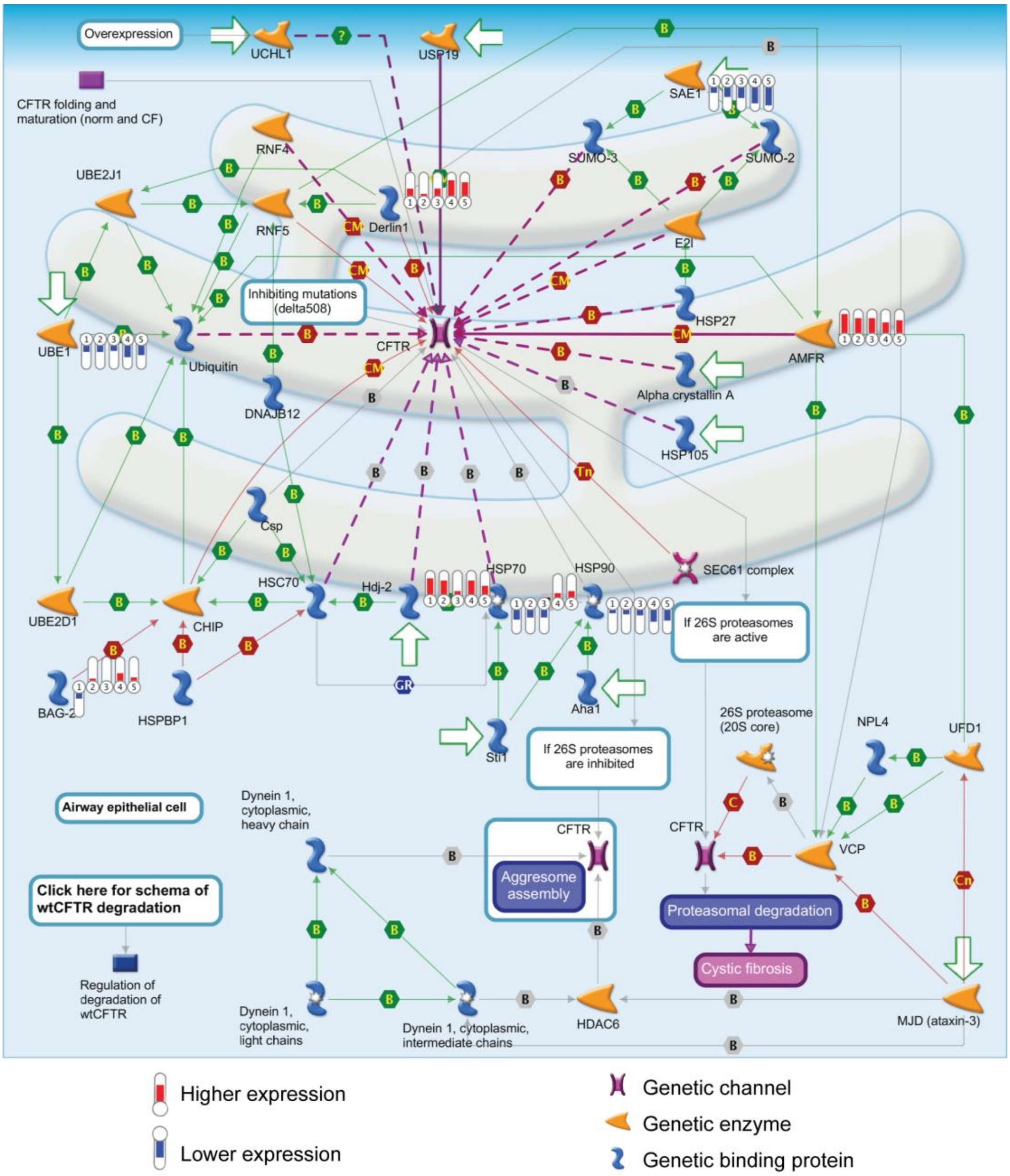

Supplement: Supplementary Information [file srep29560-s1.pdf]
